# A Comparative Analysis of Machine-Learning Algorithms for Automated International Classification of Diseases (ICD)-10 Coding in Malaysian Death Records

**DOI:** 10.7759/cureus.77342

**Published:** 2025-01-12

**Authors:** Muhammad Naufal B Nordin, Vivek J Jayaraj, Muhd Zulfadli Hafiz Ismail, Evi Diana Omar, Zamtira Seman, Yusrina M Yusoff, Najjah Tohar, Nik Noor Syamimi Ismail, Hasnah Mat, Mohd Azahadi Omar

**Affiliations:** 1 Health Informatics Centre, Ministry of Health Malaysia, Putrajaya, MYS; 2 Sector for Biostatistics and Data Repository, National Institutes of Health, Ministry of Health Malaysia, Shah Alam, MYS

**Keywords:** artificial intelligence, automation, health informatics, international classification of diseases, natural language processing, textual data analysis

## Abstract

Objective: This study explores machine learning (ML) for automating unstructured textual data translation into structured International Classification of Diseases (ICD)-10 codes, aiming to identify algorithms that enhance mortality data accuracy and reliability for public health decisions.

Methods: This study analyzed death records from January 2017 to June 2022, sourced from Malaysia's Health Informatics Centre, coded into ICD-10. Data anonymization adhered to ethical standards, with 387,650 death registrations included after quality checks. The dataset, limited to three-digit ICD-10 codes, underwent cleaning and an 80:20 training-testing split. Preprocessing involved HTML tag removal and tokenization. ML approaches, including BERT (Bidirectional Encoder Representations from Transformers), Gzip+KNN (K-Nearest Neighbors), XGBoost (Extreme Gradient Boosting), TensorFlow, SVM (Support Vector Machine), and Naive Bayes, were evaluated for automated ICD-10 coding. Models were fine-tuned and assessed across accuracy, F1-score, precision, recall, specificity, and precision-recall curves using Amazon SageMaker (Amazon Web Services, Seattle, WA). Sensitivity analysis addressed unbalanced data scenarios, enhancing model robustness.

Results: In assessing ICD-10 coding with ML, Gzip+KNN had the longest training time at 10 hours, with BERT leading in memory use. BERT performed best for the F1-score (0.71) and accuracy (0.82), closely followed by Gzip+KNN. TensorFlow excelled in recall, whereas SVM had the highest specificity but lower overall performance. XGBoost was notably less effective across metrics. Precision-recall analysis showed Gzip+KNN's superiority. On an unbalanced dataset, BERT and Gzip+KNN demonstrated consistent accuracy.

Conclusion: Our study highlights that BERT and Gzip+KNN optimize ICD-10 coding, balancing efficiency, resource use, and accuracy. BERT excels in precision with higher memory demands, while Gzip+KNN offers robust accuracy and recall. This suggests significant potential for improving healthcare analytics and decision-making through advanced ML models.

## Introduction

The death certificate is a vital public health document that indelibly records mortality details. As the foundation of mortality statistics, death records provide key personal and situational data about the deceased. They critically inform health policymaking, resource allocation, intervention evaluation, and assessments of a nation’s health and development [1,2].

By Malaysian law, all deaths must be registered with the National Registration Department (NRD). Causes of death (COD) use two sources of information: medically certified CODs validated by medical professionals or coroners and non-medically certified CODs from the police department. The Health Informatics Centre (HIC) supplies medically certified CODs, coded using the International Statistical Classification of Diseases and Related Health Problems, 10th Revision (ICD-10), by medical coders of varying coding accuracy [3]. The police department supplies non-medically certified CODs, coded using a national classification system, sometimes enhanced by verbal autopsy. The Department of Statistics Malaysia (DOSM) then compiles the dual-source COD data into official vital statistics on mortality [4].

Despite standardization efforts, up to 30% of Malaysian deaths have poorly defined causes, underscoring suboptimal death data quality and the need for refinement. Coding of textual ICD-10 data at facilities is prone to errors like misclassifications and linguistic inconsistencies, especially considering the varying experience levels of coders like doctors with many centers using more junior doctors as coders. These require linguistic processing and laborious recoding of raw text by HIC and DOSM many a time using only available bilingual short text inputs. The inaccuracies of existing methods highlight limitations in efficiently handling unstructured textual mortality data [1,2]. The hybrid system of paper-based and electronic records complicates data integration and consistency, increasing the likelihood of errors due to manual entries and insufficient cross-verification processes. Additionally, the high error rates in ICD-10 coding, despite ongoing efforts to train and retain qualified coding personnel, underscore persistent systemic challenges that hinder the reliability of health data analytics. The reliance on overburdened staff for coding accuracy not only exacerbates these issues but also leads to significant delays and potential data integrity problems, thereby complicating strategic decision-making and quality control in healthcare services [5-7]. The advancement of artificial intelligence (AI) and its subset, machine learning (ML), presents a promising avenue to address these challenges [8]. ML algorithms can process and convert unstructured textual data into standardized, structured formats [9,10]. This potential remains untapped within the local context of Malaysia's death registration system. A great deal of work has been done in the field using well-labeled electronic medical record (EMR) datasets. Studies have demonstrated the effectiveness of various ML models with large datasets, such as the Medical Information Mart for Intensive Care (MIMIC)-III, and integrated healthcare datasets from multiple institutions, showing high accuracy in disease prediction and treatment outcomes [11,12]. Nonetheless, in lower- and middle-income settings like ours, detailed EMR data may not always be available, and short bilingual textual data is often used for coding. ML can be particularly useful for this use case, providing a feasible solution to enhance the accuracy and efficiency of ICD-10 coding in resource-constrained environments.

This study aims to explore the application of ML in automating the translation of unstructured textual data from the HIC into the ICD-10 coding system. The objective is to compare various ML models, including BERT (Bidirectional Encoder Representations from Transformers) and Gzip+KNN (K-Nearest Neighbors), in automating ICD-10 coding from unstructured death records, with a focus on their feasibility and performance in resource-constrained settings.

## Materials and methods

Data

The study utilized death records from January 2017 to June 2022 obtained from the inpatient death records under the Malaysian Health Data Warehouse (MyHDW) HIC, Malaysia. The HIC houses hospital inpatient death records, coded into the ICD-10 under the Patient Treatment Information System [13]. MyHDW is currently integrated with all government facilities and some private, army, and university hospitals. The data collection procedure involved submitting a formal request to the HIC following established regulations and protocols. The data custodians pseudo-anonymized identifiable information using the Privacy Assurance Service (PAS) via the MyHDW platform, ensuring compliance with ethical standards. The inclusion criteria encompassed all death records from the MyHDW register within the specified period. Data quality checks were conducted to eliminate duplication.

Additionally, ICD-10 codes in the study had been validated each year by expert coders and certified level 2 coders through error rate studies (ERS). The study included a total of 387,650 death registrations with a short free-text structure of words in English or Malay describing the cause of death and their associated ICD-10 codes based on ICD-10, 10th revision used by MyHDW [3]. Examples of bilingual short inputs and their corresponding labels utilized in training the models are provided in Table 1. As class imbalance in ICD-10 codes is considered in the literature as an issue, we attempt to mitigate this by including all ICD-10 codes that were not present in our dataset by taking the ICD-10 codes, unrepresented in our data, as labels and their descriptions from the ICD-10 codebook as inputs.

**Table 1:** Example of short textual inputs and labels utilized for classification task comparing multiple different approaches.

ICD-10 code	Cause of death
I24.9	Heart attack
I24.9	Acute coronary syndrome with underlying rectal carcinoma with disease progression
A41.9	Blood-borne infection
A41.5	Septic shock secondary to gram-negative sepsis with acute liver failure
A97.2	Dengue shock syndrome with multi-organ failure
V89.9	Motor vehicular accident
T06.8	Severe head injury with polytrauma
J17.1	Severe COVID-19 pneumonia
V89.9	Motor vehicular accident
S09.9	Severe head injury with polytrauma

Data analysis

The dataset included only three-digit ICD-10 codes. Data cleaning procedures were applied to refine the dataset and division for training and testing using a hold-out method. Preprocessing involved several key steps to ensure data consistency and quality. HTML tags were removed using regular expressions, and tokenization was performed with Hugging Face's AutoTokenizer to convert text into a format suitable for model input. Additionally, the text was lowercased, stop words were removed, and tokens were lemmatized to standardize the textual data [14]. Pretrained models were sourced from the Hugging Face platform for the Natural Language Processing (NLP) component [15]. The BERT model, known for their efficacy in NLP tasks, was selected based on architecture, domain-specific training, model size, and parameter count [16]. The chosen models, including BERT and its variants, underwent tokenization using AutoTokenizer from the Hugging Face transformers library for efficient preprocessing. In addition to NLP models, the study encompassed a range of ML algorithms for multi-class classification, including Gzip combined with KNN, XGBoost, TensorFlow, SVM (Support Vector Machine), and Naive Bayes. BERT and its variants were included for their state-of-the-art performance in NLP tasks, leveraging bidirectional context understanding. Gzip with KNN and Naive Bayes were selected for their interpretability and computational efficiency, while XGBoost and SVM were included for their ability to handle complex, non-linear patterns and high-dimensional data, respectively. TensorFlow provided flexibility for experimenting with deep learning architectures. This diverse selection ensured robust comparisons and reliable insights.

The selection of specific ML models, including BERT, Gzip+KNN, XGBoost, TensorFlow, SVM, and Naive Bayes, was based on their established efficacy in natural language processing (NLP) tasks and multi-class classification. These models were chosen to represent a diverse range of algorithmic approaches, from traditional ML (Gzip+KNN, Naive Bayes) to advanced deep learning techniques (BERT, TensorFlow) [9,16]. Hyperparameters for each model were selected through a systematic tuning process, which involved grid search and cross-validation techniques to identify the optimal configuration for each algorithm. A more detailed discussion of hyperparameters and tuning methods for other models is provided in Table 2. Each algorithm was fine-tuned and evaluated across various parameters to establish its effectiveness in automated ICD-10 coding. The training process for each model involved consistent hyperparameters, including a maximum sequence length, batch size, training epochs, learning rate, warmup ratio, and weight decay to optimize learning and prevent overfitting. The study leveraged Python and Amazon Web Services (AWS) NIH cloud-based sandbox for comprehensive data engineering and analysis utilizing a general-purpose machine (8 CPU and 32GB memory) [17,18].

**Table 2:** Model and metric definition, mathematical formulation, text data vectorization for modelling, hyperparameter tuning and model finetuning processes . BERT: Bidirectional Encoder Representations from Transformers, KNN: K-Nearest Neighbors, SVM: Support Vector Machine.

Model/performance metric	Definition	Mathematical formulation
BERT	BERT leverages the Transformer architecture, which primarily uses the attention mechanism.	\[ \text{Attention}(Q, K, V) = \text{softmax}\left(\frac{QK^T}{\sqrt{d_k}}\right)V \]
Gzip+KNN	This equation represents the Euclidean distance metric often used in KNN for finding the nearest neighbors.	\[ d(x, y) = \sqrt{\sum_{i=1}^n (x_i - y_i)^2} \]
XGBoost	XGBoost uses a regularised learning objective that combines a traditional loss function with a regularization term that penalizes the complexity of the model.	\[ \text{Obj}(\Theta) = \sum_{i=1}^n l(y_i, \hat{y}_i) + \sum_{k=1}^K \Omega(f_k) \]
TensorFlow	In dense layers of neural networks, the output is computed as the activation function applied to the linear combination of inputs and weights plus a bias term.	\[ \hat{y} = \sigma(Wx + b) \]
SVM	SVM aims to find the hyperplane that maximizes the margin between two classes. The equation includes a regularization parameter and slack variables to handle non-linearly separable cases.	\[ \min \frac{1}{2} \| w \|^2 + C \sum_{i=1}^n \xi_i \]
Naive Bayes	Naive Bayes classifiers assume that the presence (or absence) of a particular feature of a class is unrelated to the presence (or absence) of any other feature.	\[ P(y \mid x_1, \ldots, x_n) \propto P(y) \prod_{i=1}^n P(x_i \mid y) \]
Accuracy	Measures the proportion of correct predictions (both true positives and true negatives) among the total number of cases examined.	\[ \text{Accuracy} = \frac{TP + TN}{TP + TN + FP + FN} \]
F1-Score	The harmonic mean of precision and recall, providing a balance between them.	\[ \text{F1-Score} = 2 \times \frac{\text{Precision} \times \text{Recall}}{\text{Precision} + \text{Recall}} \]
Precision	Measures the accuracy of positive predictions.	\[ \text{Precision} = \frac{TP}{TP + FP} \]
Recall	Measures the ability of a model to find all the relevant cases within a dataset.	\[ \text{Recall} = \frac{TP}{TP + FN} \]
Specificity	Measures the proportion of true negatives correctly identified.	\[ \text{Specificity} = \frac{TN}{TN + FP} \]
Precision-recall curve	A plot that displays the tradeoff between precision and recall for different threshold values. It is a useful measure of success of prediction when the classes are very imbalanced.	

Model evaluation encompassed estimating model training time, inference time, and change in memory consumption followed by evaluation of performance. Evaluation metrics included accuracy, F1-score, precision, recall, specificity, and area under the precision-recall curve (AUC-PR). Accuracy was calculated as the ratio of correctly predicted instances to the total instances. F1-score, which balances precision and recall, was computed using the harmonic mean of precision and recall. Precision was defined as the ratio of true positive predictions to the total predicted positives, while recall was the ratio of true positive predictions to all actual positives. Specificity measured the proportion of actual negatives correctly identified. AUC-PR was used to assess the trade-off between precision and recall, which is especially important for imbalanced datasets [19]. In order to estimate confidence intervals and assess the stability of our model evaluations, we employed a bootstrapping method using separate held-out test sets. This approach helps in capturing the variability and potential bias in the model performance metrics. Sensitivity analysis was also conducted on the same dataset, but without the inclusion of missing ICD-10 codes from the complete ICD-10 codebook, to evaluate the models in scenarios of unbalanced data. A detailed algorithm-by-algorithm text data vectorization, hyperparameters, model finetuning, and mathematical functions are provided in Table 3.

**Table 3:** Text data vectorization for modeling, libraries, hyperparameter tuning and model finetuning processes. TF-IDF: Term Frequency-Inverse Document Frequency, BERT: Bidirectional Encoder Representations from Transformers, KNN: K-Nearest Neighbors, SVM: Support Vector Machine.

Model	Text vectorization	Library used	Fine-tuning method	Hyperparameters
BERT	Tokenized using AutoTokenizer (Hugging Face transformers).	Hugging Face transformers	Fine-tuned with Trainer module (Hugging Face). Guided by F1 score to select the best model.	Max seq. length: 30, Batch size: 16, Epochs: 6, Learning rate: 6e-5, Warmup ratio: 0.1, Weight decay: 0.01, FP16.
Gzip+KNN	Raw text compressed using GZip for Normalized Compression Distance (NCD) calculation.	Gzip (Python Standard Library)	No training; inference uses k-nearest neighbors voting.	Number of neighbors (k): 5.
XGBoost	Text vectorized using CountVectorizer and TfidfTransformer.	Scikit-learn, XGBoost	Trained on dtrain with specified hyperparameters. Metrics saved in results directory.	Max depth: 6, eta: 0.3, Eval metric: 'mlogloss', Num class: unique train labels, Num round: 20.
TensorFlow	Vectorized with CountVectorizer and TfidfTransformer.	Scikit-learn, TensorFlow	Validation split monitored during training; metrics (accuracy, precision, F1) recorded and saved.	Layers: Dense(128), Dropout(0.5), Dense(num classes), Activation: ReLU/Softmax, Optimizer: Adam, Epochs: 10, Batch: 32.
SVM	Vectorized using CountVectorizer and TfidfTransformer.	Scikit-learn	Trained with TF-IDF vectors; metrics saved in results directory.	Kernel: 'linear', Probability estimates: Enabled.
Naive Bayes	Vectorized with CountVectorizer and TfidfTransformer.	Scikit-learn	Evaluated on TF-IDF features with metrics saved.	Default settings of MultinomialNB.

## Results

**Figure 1:**
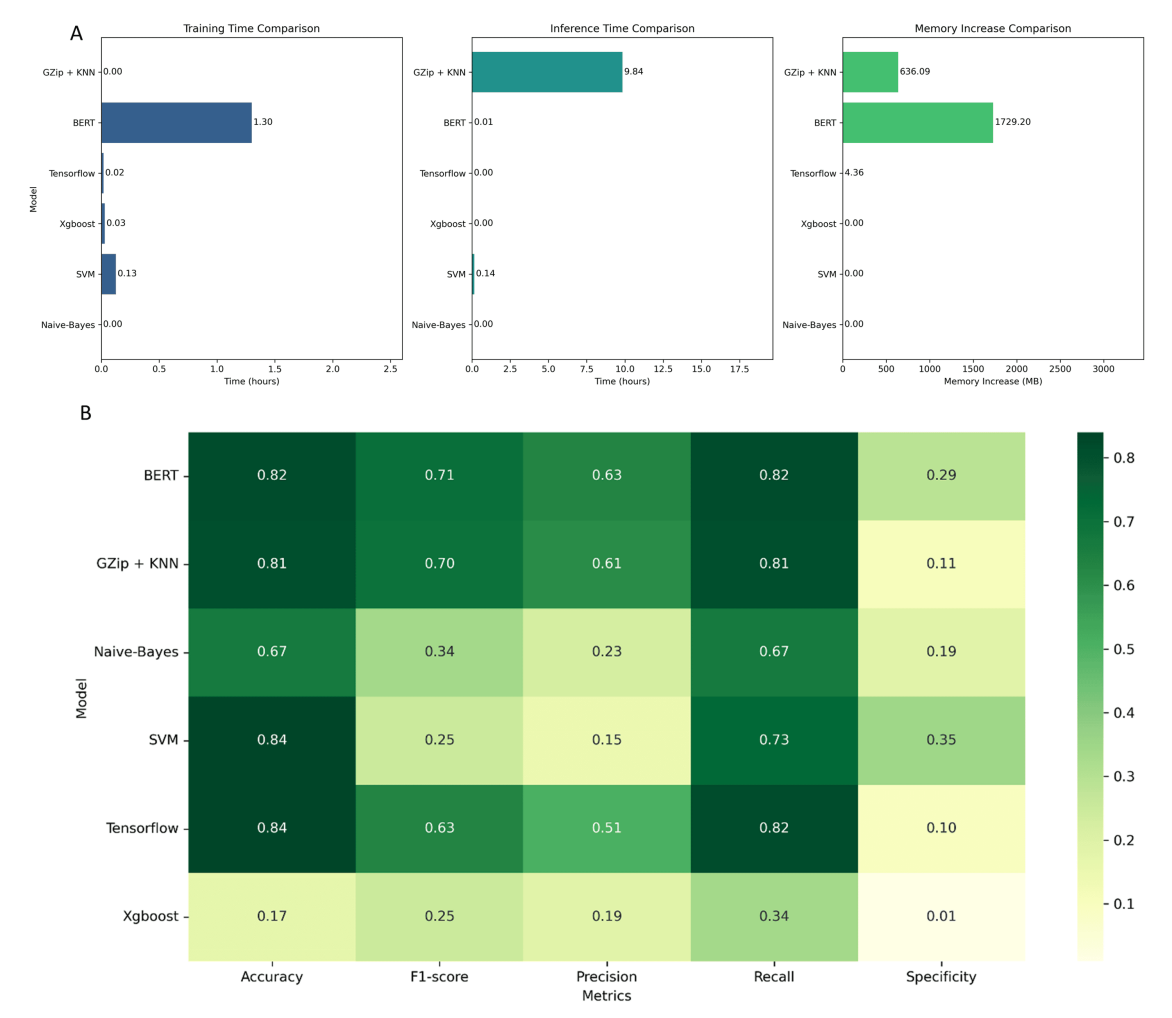
(A) Comparative analysis of model performance using training time, inference time, and memory usage for six different machine learning (ML) models utilized for ICD-10 coding, and model performance metrics. (B) Heatmap comparing accuracy, F1-score, precision, recall, and specificity across different ML models. Accuracy, F1 score, precision, recall, and specificity have been represented as mean %. Training and inference time is represented by the mean time in hours. Memory increase is represented by the mean memory use increase in megabytes. BERT: Bidirectional Encoder Representations from Transformers, KNN: K-Nearest Neighbors, SVM: Support Vector Machine.

Various ML models exhibited differential performance for ICD-10 coding. Notably, Gzip+KNN requires the longest training time, approximately 10 hours, while BERT follows with just over an hour. Memory consumption is markedly high for BERT, suggesting a considerable resource requirement (Figure 1A).

**Figure 2:**
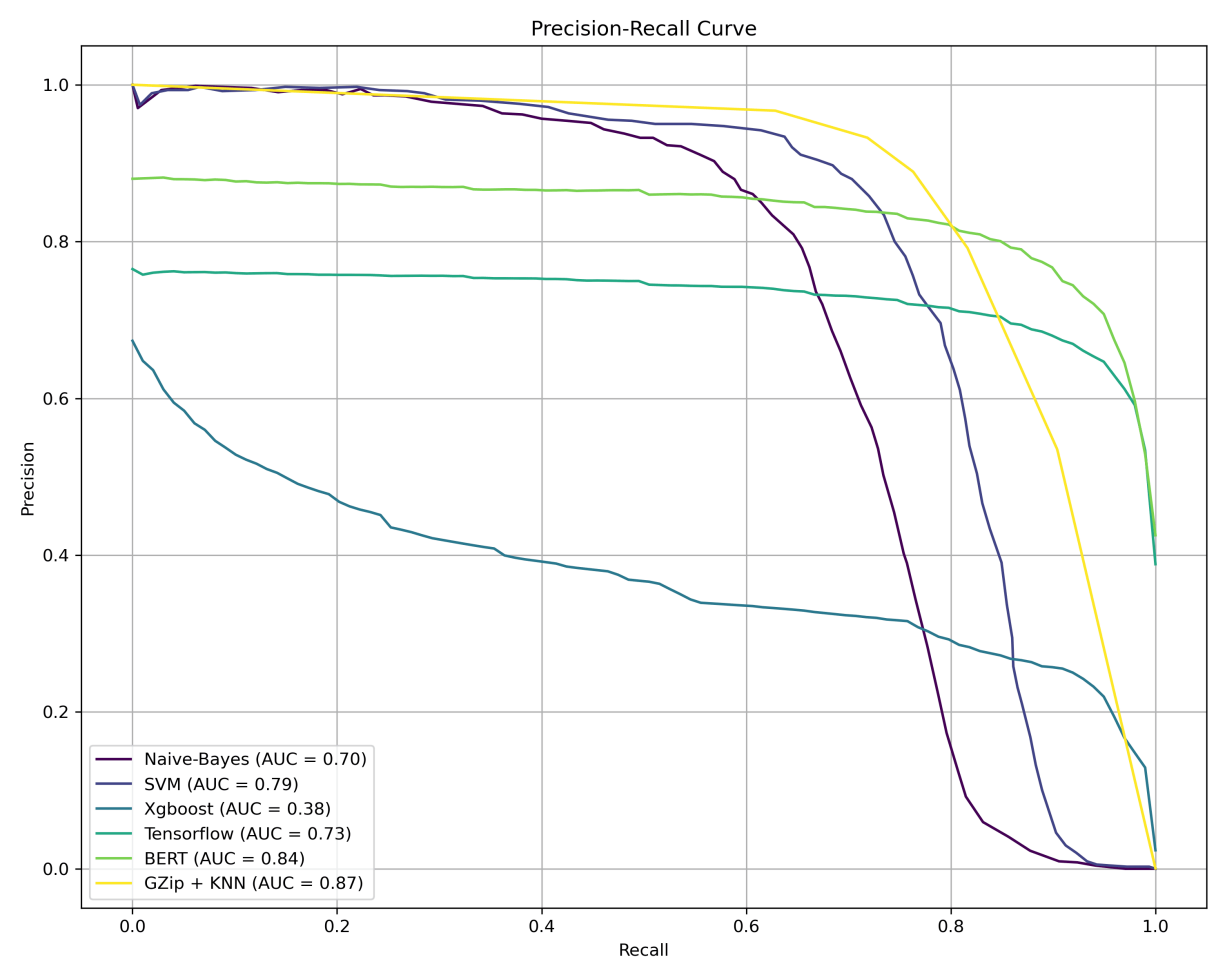
Precision-recall curves with area under the curve (AUC) for all algorithmic approaches. BERT: Bidirectional Encoder Representations from Transformers, KNN: K-Nearest Neighbors, SVM: Support Vector Machine, AUC: Area under the curve.

BERT achieved the top F1-score (0.71) and accuracy (0.82). Comparable metrics were seen for Gzip+KNN (F1: 0.70; Accuracy: 0.81). Despite a lower F1-score (0.63), TensorFlow maintained high accuracy (0.84). Notably, SVM demonstrated the highest specificity (0.35) but low F1-score (0.25). Naïve-Bayes showed moderate accuracy (0.67) and F1-score (0.34). XGBoost significantly lagged across all metrics (accuracy: 0.17; F1: 0.25). TensorFlow exhibited the highest recall (0.82) and accuracy (0.84), indicating aptitude for multi-label ICD-10 code identification. SVM also performed well with the top specificity (0.35), correctly rejecting irrelevant codes. BERT’s precision (0.63) highlights the capacity for accurate coding. Gzip+KNN showed versatility with strong accuracy, F1-score, and precision (0.81, 0.70, 0.61). Naive Bayes had lower accuracy (0.67), suggesting sensitivity-accuracy trade-offs. XGBoost’s low specificity (0.01) and accuracy (0.17) signals code exclusion and identification challenges (Figure 1B). Gzip+KNN provides the best precision-recall balance (AUC: 0.87). BERT follows closely (AUC: 0.84). SVM and TensorFlow display moderate AUCs (~0.75). Naive Bayes shows reasonable effectiveness (0.70). XGBoost trails significantly (0.38), indicating limitations (Figure 2). The estimated performance metrics and bootstrapped confidence intervals of our models are addressed in Table 4.

**Table 4:** Comparative analysis of model performance and their bootstrapped confidence intervals using training time, inference time, memory usage, accuracy, F1-score, precision, recall, and specificity for six different machine learning models utilized for ICD-10 coding. Accuracy, F1 score, precision, recall, and specificity have been represented as mean % with a 95% CI around the estimate. Training and inference time is represented by the mean time in hours with a 95% CI around the estimate. Memory increase is represented by the mean memory use increase in megabytes with a 95% CI around the estimate. BERT: Bidirectional Encoder Representations from Transformers, KNN: K-Nearest Neighbors, SVM: Support Vector Machine, AUC: Area under the curve.

	Gzip+KNN	BERT	TensorFlow	SVM
Training Time (hrs)	0 (-0.0, 0.0)	1.3 (1.3, 1.3)	0.02 (0.02, 0.02)	0.13 (0.13, 0.13)
Inference Time (hrs)	9.84 (9.84, 9.84)	0.01 (0.01, 0.01)	0 (-0.0, -0.0)	0.14 (0.14, 0.14)
Memory Increase (MB)	636.09 (624.15, 667.4)	1729.2 (1700.17, 1742.34)	4.36 (-8.12, 24.59)	0 (-21.25, 12.53)
Accuracy	0.81 (0.8, 0.81)	0.82 (0.81, 0.83)	0.84 (0.83, 0.85)	0.84 (0.84, 0.85)
F1-Score	0.7 (0.69, 0.72)	0.71 (0.7, 0.73)	0.63 (0.61, 0.63)	0.25 (0.25, 0.26)
Precision	0.61 (0.6, 0.62)	0.63 (0.62, 0.64)	0.51 (0.51, 0.53)	0.15 (0.15, 0.17)
Recall	0.82 (0.81, 0.83)	0.81 (0.8, 0.82)	0.82 (0.81, 0.83)	0.73 (0.71, 0.73)
Specificity	0.11 (0.11, 0.12)	0.29 (0.28, 0.3)	0.1 (0.09, 0.11)	0.35 (0.34, 0.36)
AUC	0.87 (0.87, 0.88)	0.84 (0.84, 0.85)	0.73 (0.72, 0.74)	0.79 (0.78, 0.8)

**Figure 3:**
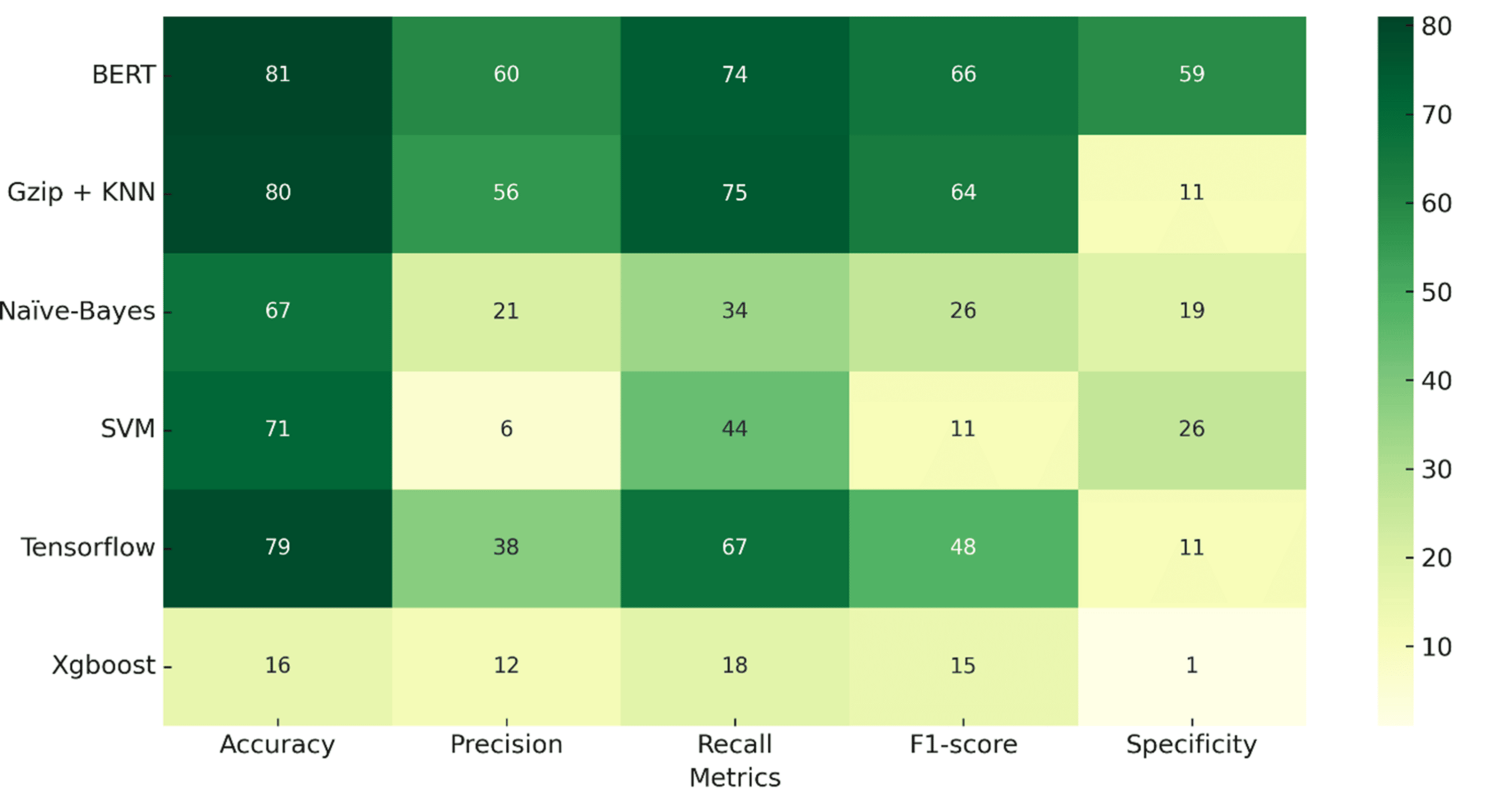
Heatmap of performance metrics from the sensitivity analysis reporting accuracy, precision, recall, F1-score, and specificity for all algorithmic approaches without the addition of missing ICD-10 codes. Note: Accuracy, F1 score, precision, recall, and specificity have been represented as mean %. BERT: Bidirectional Encoder Representations from Transformers, KNN: K-Nearest Neighbors, SVM: Support Vector Machine.

In the sensitivity analysis on the unbalanced dataset, BERT and Gzip+KNN maintained strong accuracy (~0.8), with Gzip+KNN excelling in recall (0.74) and BERT in specificity (0.59). TensorFlow also showed reasonable effectiveness. SVM, Naive Bayes, and XGBoost underperformed, particularly XGBoost, across all metrics (Figure 3).

## Discussion

The automation of ICD-10 coding from unstructured clinical death diagnosis from a national patient death register, utilizing several algorithmic approaches, revealed a complex interplay between training efficiency, resource utilization, and performance accuracy. Natural language processing using the transformer-based approach using BERT was observed to have a comparatively modest training time and higher memory usage but notable accuracy and precision, suggesting its utility in scenarios where precision is paramount. Gzip+KNN, while demonstrating the highest training duration, also displayed high accuracy and recall in both balanced and unbalanced datasets, indicating its potential to classify relevant codes accurately.

Recent literature highlights that comparative analysis of automated ICD-10 coding methodologies reveals varied performance across ML models [20-24]. In the present study, the BERT model performs most accurately with an F1-score of 0.71 and an accuracy of 82%, while Gzip+KNN closely follows, exhibiting an F1-score of 0.70. These results surpass several reported outcomes, such as Wang et al. and Chen et al., with F1-scores of 0.62 and 0.72, respectively [16,20]. However, Diao et al. report a higher F1-score of 0.88 using LightGBM, signaling the potential of gradient-boosting models in this domain [22]. Other approaches report similar F1 scores ranging from 0.65 to 0.98 across departments using CNNs, potentially suggesting large variabilities from dataset characteristics [24]. Such discrepancies underscore the need for contextual evaluation of models, considering data heterogeneity and complexity.

Relatively newer approaches such as BERT and TensorFlow demonstrated superior accuracy and F1 scores, yet their computational intensity remains a significant trade-off. Contrastingly, Gzip+KNN revitalizes traditional methods with competitive performance at lower memory costs and a simpler architecture [25]. This suggests a promising direction for future research-optimizing algorithm efficiency without compromising performance for resource-limited settings [26]. The success of such hybrid approaches underscores re-engineering established algorithms to balance efficacy and expenditure in ML applications.

Comparatively, our study addresses several unique challenges not widely covered in existing literature. For instance, while most studies, such as those using the MIMIC II and MIMIC III datasets, focus predominantly on English text, our research underscores difficulties encountered with non-English languages, which often exhibit higher error rates and complexities in coding [11,12]. This is evident in studies like Tchouka et al., which tackled French clinical texts and faced significant challenges in model training due to language-specific nuances [27]. Furthermore, unlike large dataset-based studies, our research leverages short-text data from death records, which presents unique issues such as insufficient context for accurate ML predictions [28,29]. This contrasts sharply with findings from datasets where extensive contextual data enhances model performance. Additionally, while the current trend leans heavily towards advanced NLP and deep learning models, our study also revisits the efficacy of more traditional ML approaches like Gzip+KNN. These models have shown competitive performance in our settings, highlighting their potential utility in resource-constrained environments where complex models may not be feasible.

The automated ICD-10 coding models have recently been integrated into clinical workflows in Ministry of Health Malaysia hospitals. These systems assist junior doctors in assigning standard codes during initial death data capture, overcoming limitations in manual coding competencies. Their integration serves to refine mortality statistics through more accurate structured information extraction early in the reporting process.

The analysis highlights several strengths of this study. First, the use of ML models such as BERT and Gzip+KNN demonstrated their adaptability and robustness in handling diagnostic-specific, unstructured text in a resource-limited setting. Second, this study successfully showcased the feasibility of automating ICD-10 coding, providing a pathway for streamlining manual coding processes and reducing human errors. Third, the focus on multilingual data processing, particularly for short diagnostic sentences, reflects the practical applicability of these methods in diverse healthcare settings. Lastly, the study's comparison of balanced and unbalanced datasets offers valuable insights into model behavior and performance in the presence of real-world data challenges.

There remain several limitations to this study. The scope was confined to specific models and did not encompass the entire ML spectrum. Moreover, hyperparameter tuning effects on model performance were not explored. Additionally, while the specific characteristics of our dataset, that is, very short, diagnostic-specific sentences and multilingual text primarily in the Malay language, led us to utilize all available textual content directly, this approach may limit the exploration of traditional feature selection methods that could be relevant in different or more complex textual datasets. There was limited direct applicability of advanced transfer learning techniques besides models such as BERT. Ambiguity in abbreviations like CKD (Chronic Kidney Disease) and CHD (Chronic Heart Disease) poses challenges for automated coding, as ML relies on context and training data quality. Standardized terminology and domain-specific enhancements are essential to address this limitation effectively. Additionally, despite implementing spell checks, undetected typographical errors in the dataset may affect model accuracy, representing a limitation of this study. Finally, class imbalance remains a significant challenge in training these models, as out-of-data classification remains problematic. Our findings indicated that the performance of models on balanced versus unbalanced data was fairly similar, suggesting that it is difficult to definitively assess the effectiveness of our efforts to mitigate class imbalance. Future research should consider more models, including deep learning and ensemble approaches, for a nuanced understanding of model capability [30]. Moreover, hybrid models that combine strengths could yield precise and efficient solutions [31]. The use of newer large language models within privacy-preserving environments should also be explored and is likely to have significant improvements on the methods utilized here. Such advancements could significantly contribute to biomedical semantics, improving the accuracy and utility of automated clinical coding. This analysis underscores the capabilities of various ML models in automating ICD-10 classification. It highlights balancing model complexity, resource demands, and performance. As the field progresses, integrating such technology into workflows promises to enhance data codification efficiency and reliability, thereby supporting healthcare analytics and decision-making.

## Conclusions

This study highlights that machine learning models, particularly BERT (F1-score: 0.71) and Gzip+KNN (F1-score: 0.70), effectively improve the automation process for ICD-10 coding of unstructured death records. These models demonstrate a potential balance of accuracy, resource efficiency, and robustness that could lead to advancements in healthcare analytics and decision-making by supporting current health systems’ classification of mortality data. While this study identifies these models as effective approaches, future research should include comparisons with existing manual or semi-automated coding systems to validate their efficiency and impact further, and explore the utility of hybrid approaches to optimize performance and applicability in diverse and resource-limited settings. This approach can be particularly beneficial in lower- and middle-income contexts that may not yet leverage detailed EMR systems for extracting such classifications.
